# Dysregulated miR-361-5p/VEGF Axis in the Plasma and Endothelial Progenitor Cells of Patients with Coronary Artery Disease

**DOI:** 10.1371/journal.pone.0098070

**Published:** 2014-05-27

**Authors:** Hsei-Wei Wang, Hung-Hao Lo, Ya-Lin Chiu, Shing-Jyh Chang, Po-Hsun Huang, Ko-Hsun Liao, Cheng-Fong Tasi, Chun-Hsien Wu, Tsung-Neng Tsai, Cheng-Chung Cheng, Shu-Meng Cheng

**Affiliations:** 1 Institute of Microbiology and Immunology, National Yang-Ming University, Taipei, Taiwan; 2 Genome Research Center & Cancer Research Center, National Yang-Ming University, Taipei, Taiwan; 3 Institute of Biomedical Informatics, National Yang-Ming University, Taipei, Taiwan; 4 Department of Education and Research, Taipei City Hospital, Taipei, Taiwan; 5 Department of Obstetrics and Gynecology, Hsin-Chu Mackay Memorial Hospital, Hsin-Chu, Taiwan; 6 School of Medicine, National Yang-Ming University, Taipei, Taiwan; 7 Cardiovascular Research Center, National Yang-Ming University, Taipei, Taiwan; 8 Division of Cardiology, Department of Medicine, Taipei Veterans General Hospital, Taipei, Taiwan; 9 Division of Cardiology, Department of Internal Medicine, Tri-Service General Hospital, National Defense Medical Center, Taipei, Taiwan; China Medical University, Taiwan

## Abstract

Dysfunction and reduction of circulating endothelial progenitor cell (EPC) is correlated with the onset of cardiovascular disorders including coronary artery disease (CAD). VEGF is a known mitogen for EPC to migrate out of bone marrow to possess angiogenic activities, and the plasma levels of VEGF are inversely correlated to the progression of CAD. Circulating microRNAs (miRNAs) in patient body fluids have recently been considered to hold the potential of being novel disease biomarkers and drug targets. However, how miRNAs and VEGF cooperate to regulate CAD progression is still unclear. Through the small RNA sequencing (smRNA-seq), we deciphered the miRNome patterns of EPCs with different angiogenic activities, hypothesizing that miRNAs targeting VEGF must be more abundant in EPCs with lower angiogenic activities. Candidates of anti-VEGF miRNAs, including miR-361-5p and miR-484, were enriched in not only diseased EPCs but also the plasma of CAD patients. However, we found out only miR-361-5p, but not miR-484, was able to suppress VEGF expression and EPC activities. Reporter assays confirmed the direct binding and repression of miR-361-5p to the 3′-UTR of VEGF mRNA. Knock down of miR-361-5p not only restored VEGF levels and angiogenic activities of diseased EPCs *in vitro*, but further promoted blood flow recovery in ischemic limbs of mice. Collectively, we discovered a miR-361-5p/VEGF-dependent regulation that could help to develop new therapeutic modalities not only for ischemia-related diseases but also for tumor angiogenesis.

## Introduction

It is clear that cardiovascular disorder (CVD) such as coronary artery disease (CAD), one of the most fatal causes worldwide, starts from the progressive impairment of endothelial function and integrity [1], [2]. Refurbishment of damaged microcirculation depends largely on bone-marrow-derived circulating endothelial progenitor cells (EPCs), which involve in both angiogenesis and vasculogenesis [3]–[5]. So, the dysfunction EPCs also contributes in CVD pathogenesis. Reports showed that the number of EPCs is inversely correlated to progression of coronary heart disease [6]. Levels of EPCs are also negatively correlate with angiogenesis-related phenotypes such as diabetes mellitus and aging [5]. On the other hand, angiogenic activities in patients are also reduced. Patients at cardiovascular risk with both low EPC counts and impaired EPC activities have a higher incidence for cardiovascular events and higher mortality [7]. There studies also lead to the rationale for autologous stem cell therapy in different clinical settings (www.clinicaltrials.gov/) [2].

One of the mechanisms responsible for the reduced EPC amount and activities in the systemic circulation of patients may due to the reduced expression of angiogenic cytokines such as CXCL12 (stromal cell-derived factor 1, SDF1) and VEGF in patients [8], [9]. SDF1 acts as a chemoattractant for EPCs and suppresses their apoptosis, while mitogenic activity of is limited [10]. VEGF is a known EPC mitogen, which increased the proliferation rate of EPCs as well as the activities. After mobilization from bone marrow, reduced VEGF levels may result in the slow growth and suppressed the activation of circulating EPCs. *In vitro* cellular studies and preliminary animal results suggested that VEGF gene transfer would augment EPC proliferation, adhesion, incorporation into endothelial cell monolayers, and *in vivo* neovascularization [11]. In the patients with critical limb ischemia receiving VEGF gene transfer, levels of circulating endothelial progenitor cells were amplified [11].

Currently, EPCs can be isolated from adult peripheral blood (PB) and umbilical cord blood (CB) [12], yet the number of EPCs could be obtained in adult blood is significantly lower than that in cord blood [13]. It has been shown that EPCs from cord blood have superior vasculogenic ability, evidenced by their higher proliferative advantage and better survival rate in vitro [14]. Also, blood vessels formed by adult peripheral blood EPCs (PB-EPCs) are unstable and regress within weeks, while those from umbilical cord blood EPCs (CB-EPCs) function normally blood and can last for more than 4 months [15]. We further weighted in this field by showing that cord blood EPCs have better cell migration and microtubule formation activities than those from PB [12]. Mechanistically, we showed by small RNA sequencing that angiogenic microRNAs (miRNAs, miRs) such as miR-31 are more abundant in CB-EPCs. Overexpressing miR-31 in PB-EPCs helped to increase cell migration and microtubule formation functions [12].

The above example illustrates the critical regulatory role of microRNAs in EPC biology. In the human genome, more than 2500 miRNAs have been discovered (according to the miRBase 20 release). Tissue miRNAs, as well as the circulating ones in body fluids, have recently been considered as a potential approach to identify new disease biomarkers and develop novel drug targets [16]. Regarding VEGF regulation, several miRNAs have been reported to suppress VEGF expression [17]. Vascular endothelial cell-specific microRNA-15a directly targets FGF2 and VEGF to inhibit angiogenesis [18]. microRNA-195 suppresses angiogenesis and metastasis of hepatocellular carcinoma by inhibiting the expression of VEGF and CDC42 [19]. Nevertheless, which miRNAs may suppress VEGF levels in diseased EPC to contribute to CAD pathogenesis remains unexplored. Here we hypothesized that the lower VEGF level in the diseased EPCs might be due to abnormally abundance of anti-VEGF microRNAs inside cells. By comparing the miRNA expression profiles between various EPCs with distinct angiogenic activities, we uncovered a significant miR-361-5p/VEGF axis for regulating EPC activities. To expand the clinical relevance and significance, we also examined the level of miR-361-5p in plasma from diseased individual to evaluate the potential of being a novel diagnostic biomarker for coronary artery disease.

## Materials and Methods

### Ethics Statement

This study was approved by the institutional review boards of the Tri-Service General Hospital and the Taipei Veteran General Hospital (IRB number: 2012-03-001AC). All participants provide their written informed consents to participate in this study. The protocols of this study are consistent with the ethical guidelines of the 1975 Helsinki Declaration. All experimental procedures and protocols involving animals were approved by the Institutional Animal Care and Use Committee (IACUC) of National Yang-Ming University and in compliance with the ARRIVE guidelines.

### Isolation and characterization of peripheral blood EPCs from CAD patients and healthy donors

EPCs were isolated and collected from peripheral blood samples from healthy donors or CAD patients. Patients who diagnosed with Coronary Artery Disease by Cardiac Catheterization were considered to be the criterion for CAD patients and vice versa. The baseline characteristics of studied subjects in healthy and patients were listed in the *Table S2* online. In brief, mononuclear cells (MNCs) were isolated by Histopaque-1077 (1.077 g/ml, Sigma, St. Louis, MO, USA) by density centrifugation. The MNCs were further resuspended in 2 ml endothelial growth medium-2 (Lonza Ltd, Basel, Switzerland) with complete supplements (Hydrocortisone, IGF-1, hEGF, hVEGF, hFGF2, ascorbic acid, GA-1000, heparin and 2% FBS) and seeded to the fibronectin-coated plate for cultivation. After 14–21 days of culturing, attached late EPCs appeared to be cobblestone-shaped and monolayer growth pattern typical of mature endothelial cells at confluence.

These late EPCs, which exhibited both endothelial and hematopoietic stem cell surface markers as described [12], were introducing for all experiments in this study. For avoiding the aging issue or the senescence of the primary cell culture, all the EPCs for functional analysis and RNA extraction will not be cultured if exceed six passages. The ideal culture condition followed manufacture's instructions faithfully.

### Small RNA sequencing (smRNA-seq) data analysis

Global miRNA profiles of cord blood (CB) and peripheral blood (PB) EPCs were from our previous study [12], and results were analyzed by a published bioinformatics pipeline constructed in our lab [20]. Fastq raw sequences, which were without poly-A tracts, ambiguous nucleotides or a 5′ adapter, but contained the flanking 6–18 nt of the 3′ adapter sequence, had the adapter sequence trimmed and the identical sequences were then collapse to a series of unique sequences. As to miRNA quantification, all pre-processed datasets were mapped to the miRNA list based on miRBase R19 using Bowtie with options: -a -v 1 -S –f –norc, and then alignment results were produced in a BAM file format by SAMtools [20]. The BAM files were processed by in-house JAVA software for miRNA quantification [20]. Expression values of specific miRNAs were calculated as the RPM value (Reads Per mapped reads), namely RPM = C/MN×10^6^, where C is "read numbers aligned to a given miRNA chromosomal region”, M is “multiple mapping numbers across all miRNA regions by one read (i.e., the number of different miRNA chromosome locations mapped by the same read)” and N is “total read numbers that map to human genome sequence”. To minimize the effect of cross mapping of sequences with uncertain genomic locations, the expression is divided by its number of cross-mapping events.

### microRNA quantitative RT-PCR

RNA extraction and RT-qPCR were performed as described [12]. Total RNA was extracted using Tri Reagent (Sigma-Aldrich Co., St. Louis, USA) according to the manufacturer's instructions. For miRNA qPCR, the expression levels of specific miRNAs were detected using stem-loop RT-PCR [21]. The universal PCR reverse primer for the miRNAs was 5′-GTGCAGGGTCCGAGGT-3′. Amplification of marker genes was performed using specific primers, Maxima SYBR Green qPCR Master Mix (K0222, Fermentas) and a StepOne sequence detector (Applied Biosystems, USA). Designed primers for each miRNAs are listed in *Table S1*; endogenous U6 and miR-16 were used as an internal control for normalizing miRNA expression in cells and plasma respectively [12]. The VEGF mRNA expression data were normalized to the average value of GAPDH and beta-actin.

### Transfection of microRNA oligonucleotides and plasmids

The lentiviral expression vector expressing miR-361-5p precursor sequence was constructed using the following primer pairs: forward: 5′-TTgggCATATgTgACCATCA-3′; reverse: 5′-ggAgCTCAACCATACCAggA-3′. Both micr*ON* agomir and micr*OFF* antagomir (RiboBio Co., Guangzhou, China) for miR-361-5p or miR-484 are commercial synthetic RNA molecules with several chemical modifications for direct transfection without transfect reagents. To over-express or knock down miR-361-5p and miR-484 in EPCs, agomir or antagomir were added into culture medium at a concentration of 50 nM at 70% to 80% cell confluence. Both agomir and antagomir are stable in EPC for at least 14 days for further *in vitro* or *in vivo* studying (not shown). The expression level of transfected microRNAs were monitored and measured by quantitative RT-PCR.

### EPC tube formation, transwell cell migration and cell proliferation assays

*In vitro* tube formation assay was performed on EPCs for assessing the capacity of neovascularization as described [12]. Thawed Basement Membrane Extract (BME, 3433-005-01, Trevigen Inc.) were plated in 96-well at 37°C for up to 1 hour to form a reconstituted basement membrane. EPCs, less than six passage of cultivation, were collected by trypsin/EDTA, and 1×10^4^ cells in 100 µl medium were seeded on Matrigel then incubated at 37°C for 6 hours. Tube structures were inspected under an inverted light microscope (100 X). To evaluate the tube formation capacity, five representative fields were captured and analyzed by calculating total tube length in each group. All data were obtained from three independent experiments with triplication. For easily interpret the significance of the experiments, the total tube length were further normalized to control group and presented into relative tube length.

For Transwell cell migration assay, 600 µl medium with 10% FBS were added to the lower chamber, while 5×10^4^ EPCs in 100 µl medium were subjected to upper chamber of Costar Transwell Polycarbonate Permeable Supports (Corning, NY, USA). After 3 hours incubation at 37°C, cell suspensions were removed from upper chamber and the 8 µm permeable membranes were fixed with 4% paraformaldehyde for at least 15 minutes at room temperature. Migrated cells were then stained with Hochest 33342 reagents (Sigma-Aldrich) for 30 minutes and counted under fluorescent microscope by five representative fields. The degree of cell proliferation was examined by the MTT assay system (Invitrogen, USA) according to the manufacturer's instructions.

### Reporter assays

For luciferase reporter plasmids, the predicted microRNA-binding site was cloned into the XbaI site of the pGL3-Basic plasmid (Promega, Wisconsin, USA) with the following primers: VEGF-UTR-F, 5′-CCgTCTAgATCTTTTgCTCTCTCTTgCTCTC-3′; VEGF-UTR-R, 5′AgCTCTAgAACggATAAACAgTAgCACCAA-3′. The luciferase reporter plasmids containing VEGF mutant binding site was created using the QuikChange Site-Directed Mutagenesis Kit (Stratagene, USA). For 3′UTR reporter assays, miRNA and reporter plasmids were co-transfected by the lipofectamine 2000 reagent (Life Technologies) into 293T cells and analyzed by the measurement of ratio between firefly and rellina luciferase activities.

### Mouse ischemic hindlimb model and EPC transplantation

Nude mice ranging from 6 to 8 weeks were purchased from the National Laboratory Animal Center (Taiwan) and kept in microisolator cages on a 12-h day/night cycle for 2 weeks before operation. After two-week stabilization, mice received right femoral artery excision for inducing unilateral hindlimb ischemia as previously described [22]. Briefly, mice were anesthetized by intraperitoneal injection of ketamine (100 mg/kg) and xylazine (10 mg/kg). Both proximal and distal portion of right femoral artery were ligated, as well as distal portion of saphenous artery. Mice were randomly allocated to three groups (n = 6) with different treatments: EGM2 medium, CAD-EPC with scramble oligonucleotides, and CAD-EPC with miR-361-5p antagomirs (micrOFF, RiboBio Co., Guangzhou, China). CAD-EPCs from same donors were pre-stained with PKH26 (Sigma-Aldrich), a tracking dye for staining cell membrane, before transplantation. After 72 hours, a total volume of 200 µl medium with 2.5×10^5^ EPCs were injected intramuscularly at six different sites of ischemic limb distal to the arterial occlusion site. Blood perfusion was monitored by Laser Doppler Perfusion Imager (LDPI) system (Moor Instruments Limited, Devon, UK) before and after the surgery, and was then measured weekly. To prevent individual difference, the results were indicated as the ratio of perfusion in the ischemic (right) versus non-ischemic (left) limb.

### Statistical analyses

All data were presented as mean ± the standard deviation as indicated in each graph. The results were statistically analyzed using the software GraphPad Prism 5. To examine the effects of molecular manipulation, in control and experimental groups, *Student's* T test was applied to compare and determine the significant difference (such as gene overexpression and knockdown). One-way ANOVA test followed by Tukey's post-hoc test was applied to determine the significance among each group of luciferase reporter assay and all double manipulation assay with more than two sample groups. Mann-Whitney *U* test was used to analyze the significance of gene expression, circulating microRNAs level and VEGFA ELISA in healthy donors or CAD patients in EPCs and plasma, respectively. The rescuing effects of miR-361-5p antagomirs to the angiogenic activities of CAD-EPC were evaluated by a one-way ANOVA test followed by Tukey's post-hoc test. Recovery of blood flow, capillary densities and CD31/PKH-26 double positive cell counts were conducted to a one-way ANOVA test followed by Tukey's post-hoc test. A *p*-value that is lower than 0.05 was considered statistically significant.

## Results

### Isolation and characterization of human EPCs from the peripheral blood of CAD patients and healthy subjects

**Figure 1:**
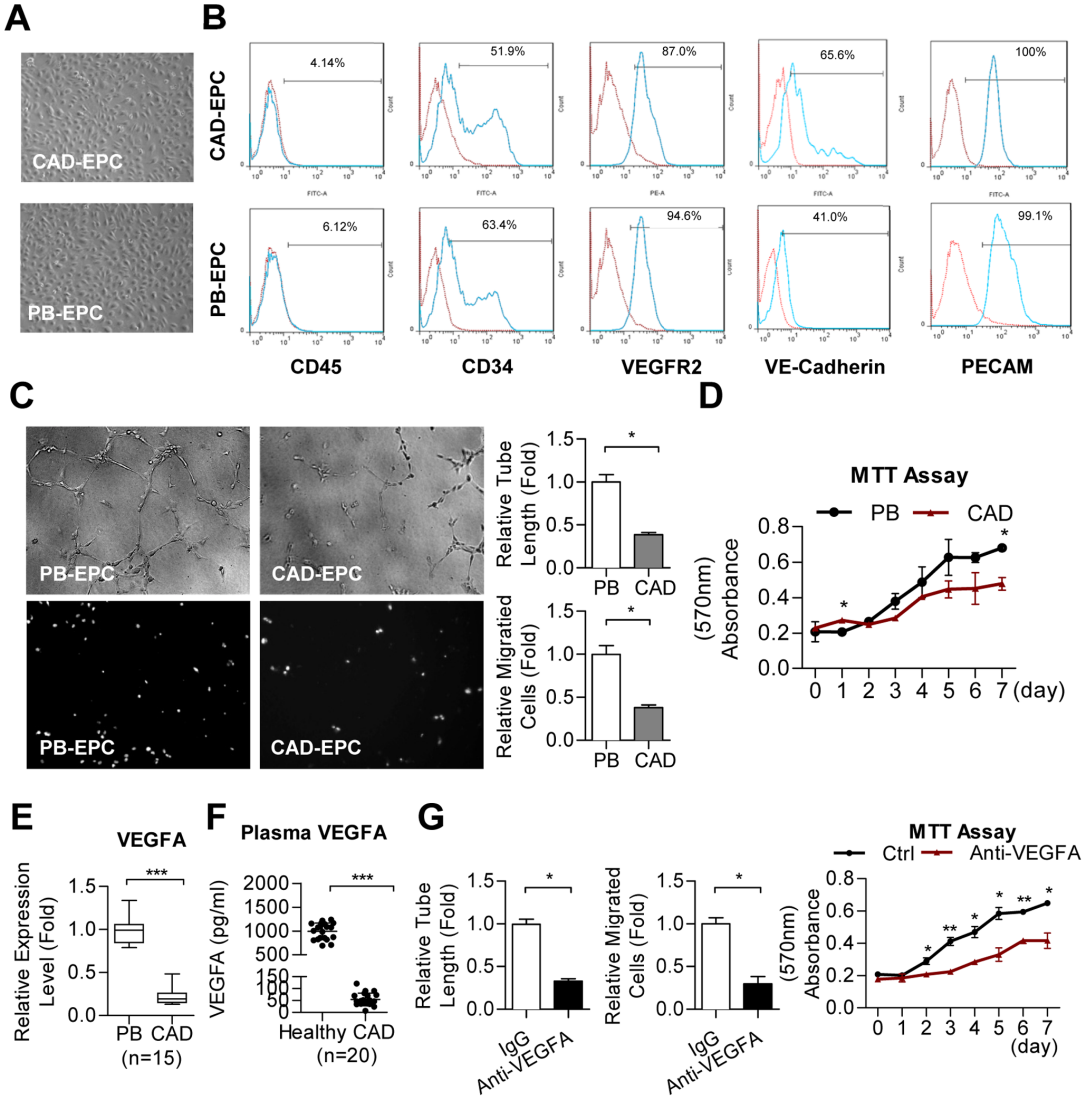
Reduced VEGF levels and angiogenic activities in EPCs from CAD patients. (**A**) Morphology of healthy and diseased EPCs. (**B**) Expression of indicated molecules in EPCs by flow cytometric analyses. (**C**) Different angiogenic abilities between healthy and diseased EPCs. EPCs from peripheral blood of healthy individuals (PB-EPCs) migrate faster and form better microvasculature structures *in vitro* than those from patients with CAD (CAD-EPCs). EPCs from different sources were subjected to Transwell cell migration assays or onto MatriGel for tube formation assays. Migrated cells were stained (representative pictures are shown, left lower panel) and counted (right panel, n = 3). Tube lengths of formed microvascular structure were also measured (middle panel, n = 3). *: *p*<0.05 by *Student's* T test. (**D**) Cell proliferation assays show PB-EPCs grow faster in vitro. Cultured EPCs were subjected into MTT assays for monitoring cell proliferation rate. *: *p*<0.05 by *Student's* T test. (**E**) Hierarchical VEGF mRNA expression levels in CAD-EPC and healthy PB-EPC showed by RT-qPCR. Mean gene expression levels of EPC genes were compared to the average CT values of GAPDH and beta-actin controls. Results are expressed as mean±standard deviation. ***: *p*<0.001 by Mann-Whitney *U* test. (**F**) Reduced serum VEGF protein levels in CAD patients determined by ELISA assays. ***: *p*<0.001 by Mann-Whitney *U* test. (**G**) Treating PB-EPCs with the Avastin anti-VEGF mAb suppresses *in vitro* microtubular formation, cell migration, and cell proliferation activities. *: *p*<0.05, **: *p*<0.01 by *Student's* T test.

EPCs with a cobblestone-like morphology similar to mature endothelial cells were obtained from peripheral blood of CAD patients (CAD-EPCs) or healthy subjects (PB-EPCs) as described (Fig. 1A) [23]. FACS analyses showed that both EPCs were negative for the hematopoietic marker CD45 yet the CD34 precursor gene (Fig. 1B). They also expressed endothelial markers VEGFR2, VE-cadherin, and PECAM (CD31) (Fig. 1B).

Cultured EPCs were subjected into Transwell cell migration and tube formation assays, and clearly PB-EPC migrated faster and formed microvasculature structure more efficient than diseased EPCs (Fig. 1C). PB-EPCs also proliferate faster than CAD-EPCs (Fig. 1D). Levels of VEGF transcripts in different EPCs inversely correlated with their angiogenic activities (Fig. 1E). Furthermore, ELISA data confirmed that VEGF protein levels in the plasma of CAD patients were lower than those in healthy controls (Fig. 1F). The critical function of VEGF on EPC activities was confirmed by adding the anti-VEGF mAb (Avavstin) into culture medium of PB-EPCs. Cell motility, microvasculature formation ability, and cell proliferation activity of treated EPCs were all reduced significantly (Fig. 1G).

### Candidate anti-VEGF miRNAs in CAD-EPCs and patient circulation

**Figure 2:**
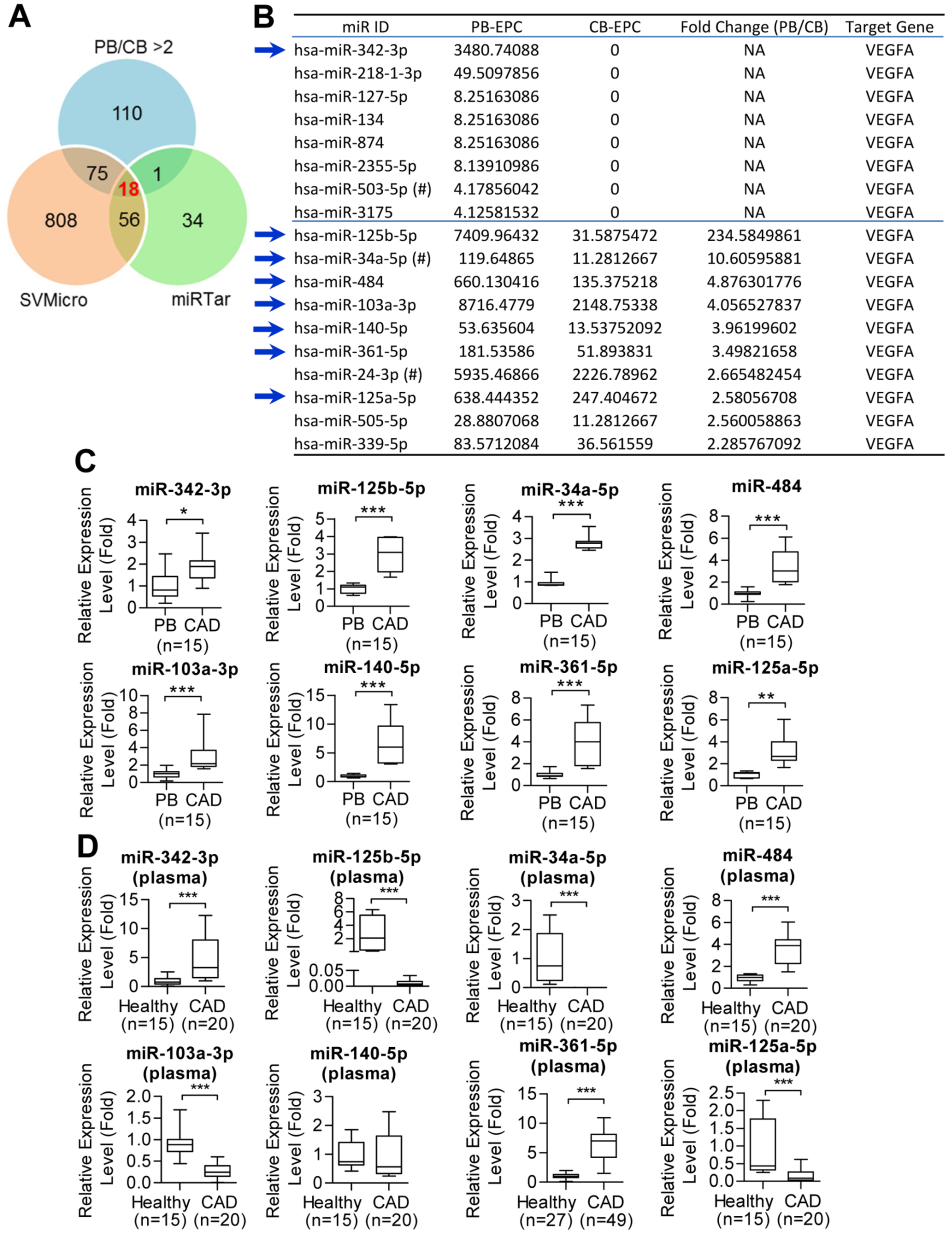
smRNA-seq reveals candidate anti-VEGF miRNAs in both EPCs and the plasma of CAD patients. (**A**) A Venn diagram showing overlapping miRNAs between smRNA-seq data and bioinformatics prediction results. (**B**) A table highlighting candidate VEGF-targeting miRNAs highly expressed in PB-EPCs. #: miRNAs known to target VEGF directly; arrows: miRNAs picked for further RT-qPCR validation. (**C–D**) RT-qPCR showing expression levels of 8 miRNAs in EPCs (*C*) and the plasma (*D*) of normal controls (PB) and CAD patients. *: *p*<0.05; **: *p*<0.01: ***: *p*<0.001 by Mann-Whitney *U* test.

We hypothesized that miRNAs are involved in regulating VEGF expression, hence lead to the discrepancy of angiogenic activities in different EPCs. We explored miRNA expression patterns of different EPCs by small RNA sequencing (smRNA-seq), hypothesizing that miRNAs targeting VEGF must be more abundant in EPCs with less activities. Our previous study deciphered the miRNA expression profiles of cord blood EPCs (CB-EPCs) and healthy PB-EPCs, in which we also found CB-EPCs proliferated more rapidly, migrated faster and formed tubule structure more efficiently than PB-EPCs [12]. According to our own published smRNA-seq results, we found a total of 204 miRNAs with more than 2-fold changes were more abundant in PB-EPCs (Fig. 2A). Two bioinformatics algorithms, miRTar [24] and SVMicro [25], were applied to filtrate anti-VEGF miRNAs from these 120 miRNAs, and 18 candidates were identified by both algorithms (Figs. 2A–B). Among them, miR-34a inhibits tumor angiogenesis by blocking autocrine VEGF production [26], and levels of miR-34a were higher in CAD-EPCs than in control EPCs [27]. MicroRNA-24 has been proved to target VEGF mRNA directly [28], and miR-24 is also loss in the plasma and of type 2 diabetes patients [29]. Furthermore, miR-503, whose expression is down-regulated by hypoxia through HIF1α, also targets FGF2 and VEGFA for inhibiting tumor angiogenesis and growth [30]. Deregulation of microRNA-503 contributes to diabetes mellitus-induced impairment of endothelial function [31]. These consistent data suggested that our genomics approach hold the potential for the identification of novel anti-VEGF microRNAs.

We started validating *in silico* data by detecting the levels of candidate miRNAs in EPCs. All eight miRNAs examined (indicated by arrows) showed significant upregulation in CAD-EPCs (Fig. 2C). Further exploration of their levels in the plasma of CAD patient and control cases, only circulating miR-361-5p and miR-484 were more abundant in CAD cases by RT-qPCR (Fig. 2D). Levels of circulating miR-140-5p showed no different between healthy and disease population (Fig. 2D). Moreover, levels of miR-342-3p, miR-125b-5p, miR-34a-5p, miR-103a-3p, and miR-125a-5p were even reduced significantly in patient circulation (Fig. 2D).

### miR-361-5p, but not miR-484, regulates EPC function *via* targeting VEGF

**Figure 3:**
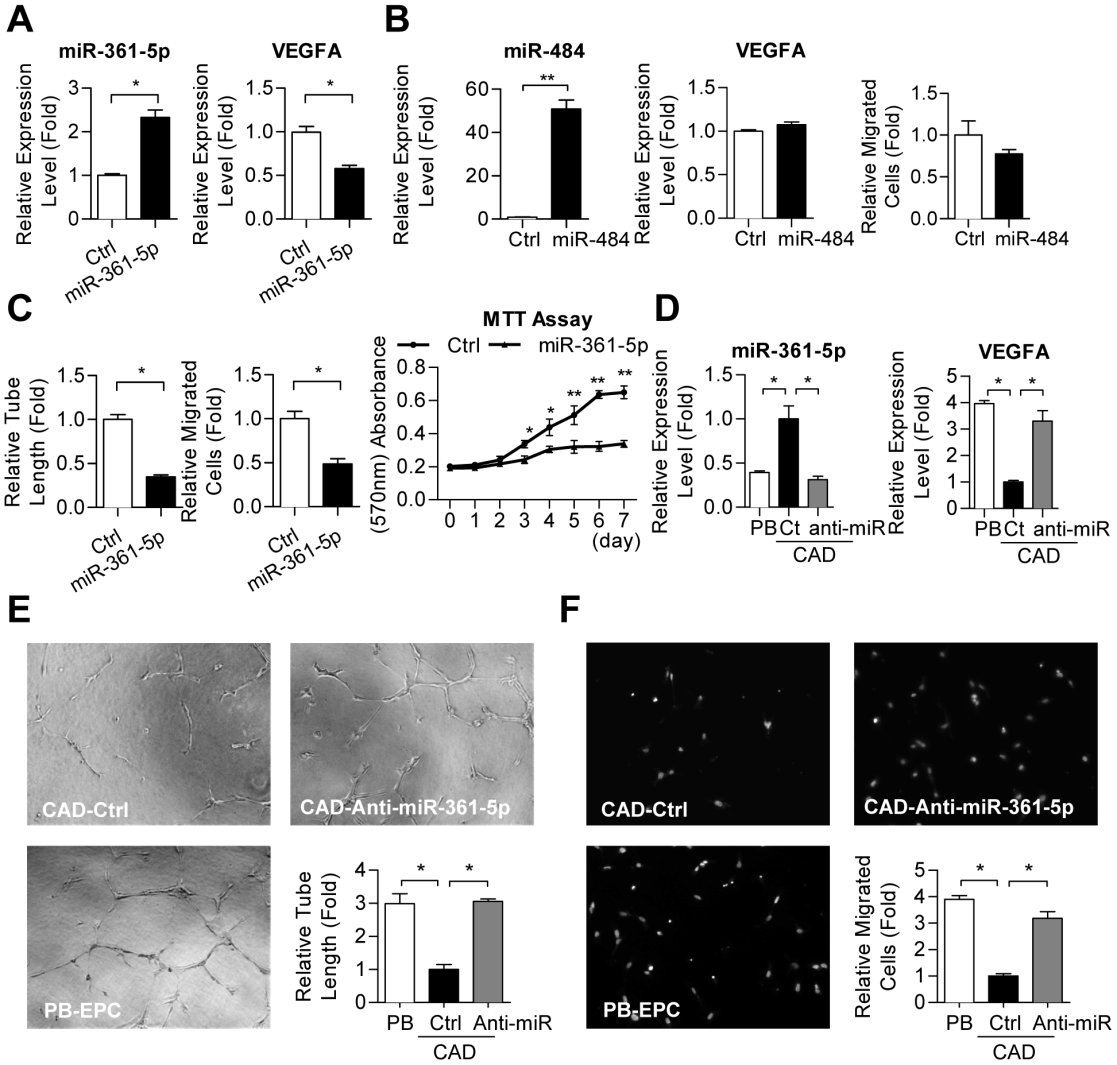
Suppression levels of miR-361-5p, not miR-484, restored VEGF expression and EPC functions. (**A**) Overexpression of miR-361-5p in PB-EPCs down-regulated VEGF levels. *: *p*<0.05, by *Student's* T test. (**B**) Overexpression of miR-484 in PB-EPCs neither down-regulated VEGF levels (*middle panel*) nor inhibited cell motility (*right panel*). **: *p*<0.01 by *Student's* T test. (**C**) miR-361-5p transfectants showed reduced microtubule formation (left), cellular migration (*middle*), and cell proliferation (*right*) abilities. Representative pictures are shown in *Suppl.* Fig. 1 online. *: *p*<0.05, **: *p*<0.01 by *Student's* T test. (**D–F**) Restoring miR-361-5p level in CAD-EPCs repairs EPC functions. (*D*): RT-qPCR shows that miR-361-5p oligonucleotide antagomir transfection represses CAD-EPC miR-361-5p to a level similar to that in normal PB-EPC controls. EPC vasculogenesis (*E*) and migration (*F*) abilities were also measured. *: *p*<0.05 by one-way ANOVA test followed by Tukey's post-hoc test.

We then investigated the effects of miR-361-5p and miR-484 on VEGF levels and EPC activities. Overexpression of miR-361-5p, but not miR-484, resulted in the reduction of VEGF transcripts in healthy EPCs (Figs. 3A–B). Consistently, EPC motility was only inhibited in PB-EPCs transfected with miR-361-5p, but not those with miR-484 (Figs. 3B–C and Fig. S1A). Consistently, microvasculature formation and cell proliferation abilities of miR-361-5p transfectants were also reduced significantly (Fig. 3C and Fig. S1A). Knock down of miR-361-5p, but not miR-484, in CAD-EPCs to a level compatible to that in healthy EPCs by oligonucleotide antagomirs restored VEGF expression (Fig. 3D and Fig. S1B). More importantly, angiogenic-related abilities, including the migratory and microtubule formation activities, of diseased EPCs were rescued to levels similar to those of healthy controls (Figs. 3E–F).

**Figure 4:**
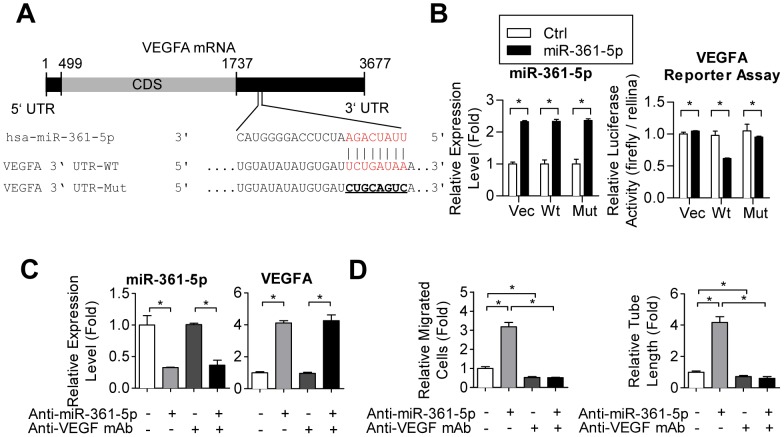
The miR-361-5p/VEGF pair contributes to CAD-EPC activities. (**A**) Structure of the VEGF transcript and the predicted binding site between the VEGF 3′UTR (untranslated region) and miR-361-5p. (**B**) A luciferase reporter plasmid containing either the miR-361-5p binding site (WT) or a mutant (Mut) binding site were co-transfected with miR-361-5p. The luciferase activity was assessed 2 days post-transfection (*right panel*). The expression levels of miR-361-5p were detected by RT-qPCR (*left panel*). *: *p*<0.05 by one-way ANOVA test followed by Tukey's post-hoc test. (**C–D**) Neutralization of VEGF in miR-361-5p-reduced (by antagomirs) CAD-EPCs suppresses cellular activities. miR-361-5p and VEGF levels in treated CAD-EPCs were determined by RT-qPCR (*C*). Transwell migration (*D, left panel*) and tube formation (*D, right panel*) assays were conducted on CAD-EPCs treated with the indicated antagomirs and/or the Avastin anti-VEGF mAb. Representative pictures are in *Suppl.* Figs. 2A-B online, and the quantitative results of the images are shown. *: *p*<0.05 by one-way ANOVA test followed by Tukey's post-hoc test.

The direct targeting of VEGF by miR-361-5p was explored by luciferase reporter assays, and it was found that miR-361-5p repressed luciferase expression when the construct contained the VEGF 3′UTR fused downstream of the luciferase gene (Fig. 4A–B, the UTR-WT group). Such repression could be reversed by mutating the predicted miR-361-5p -binding site (Fig. 4A–B, the UTR-Mut group).

To further clarify the hierarchical relationship between miR-361-5p and VEGF, we compared the results of knocking down miR-361-5p to those of blocking VEGF activities (by adding the Avastin anti-VEGF mAb), either independently or in combination with miR-361-5p knockdown. CAD-EPCs with reduced miR-361-5p showed higher VEGF expression, better cellular motility, and superior tube formation ability (Fig. 4D, lanes 1 *vs*. 2 & Figs. S2A–B; miR-361-5p levels detected by RT-qPCR were in Fig. 4C), whereas diminishing VEGF activities in miR-361-5p^low^/VEGF^high^ CAD-EPCs abolished angiogenesis-related activities (Figs. 4C–D, lanes 2 vs. 4 & Figs. S2A–B). Of note, blocking VEGF activity did not affect the expression of miR-361-5p (Fig. 4C), and the addition of recombinant VEGF on CAD-EPCs did not either (Figs. S2C). These findings suggested that VEGF is an important direct downstream target of miR-361-5p, and the regulation of miR-361-5p/VEGF axis is unidirectional but not reciprocal.

### miR-361-5p promotes blood flow recovery in ischemic limbs in mice

**Figure 5:**
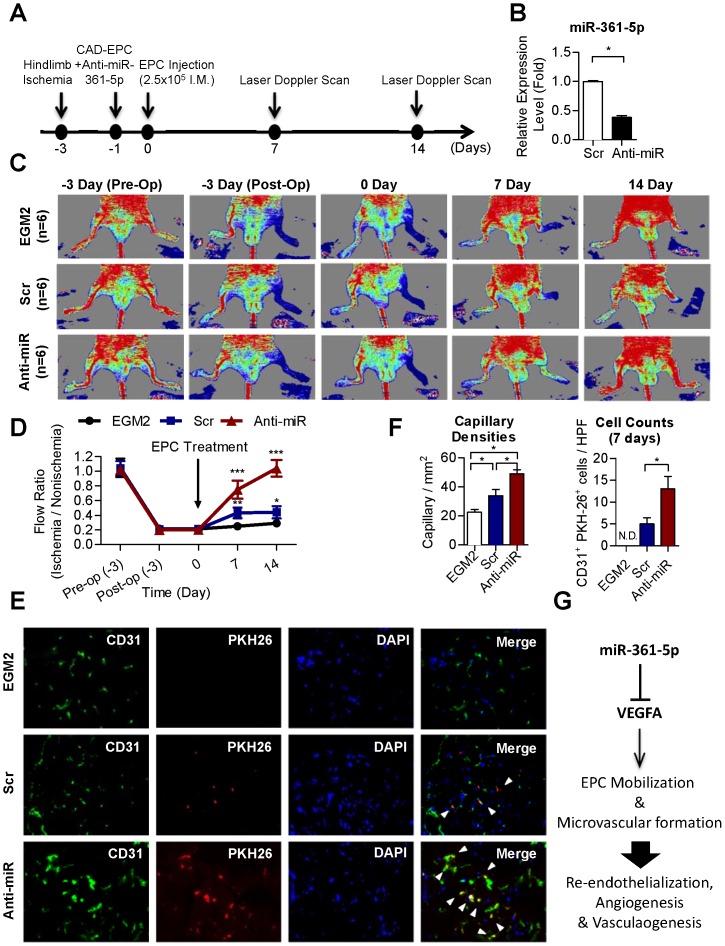
Transplantation of miR-361-5p^low^ CAD-EPCs improves blood perfusion in the ischemic hindlimb. (**A**) Schematic represeatation of experimental design. (**B**) miR-361-5p levels in tranfected CAD-EPCs determined by RT-qPCR. *: *p*<0.05 by *Student's* T test. (**C**) Representative images of hindlimb blood flow measured by laser Doppler before operation (Pre-Op), immediately after hindlimb ischemia surgery (Post-Op), and 2 weeks after intramuscular injection of culture medium (EGM2), peripheral blood EPC-transfected with scramble oligonucleotides (Scr), or CAD-EPC-transfected with miR-361-5p oligonucleotide antagomirs (Anti-miR). (**D**) Quantitative analysis of blood flow expressed as perfusion ratio of the ischemic to the contralateral (non-operated) hindlimb. *: *p*<0.05; **: *p*<0.01: ***: *p*<0.001 compared with control; n = 6. (**E**) Immunofluorescence staining on nude mice tissues 7 days after injection with PKH-26-labeled CAD-EPCs. Capillaries in the ischemic muscles were visualized by anti-CD31 immunostaining (green), and injected human EPCs were monitored by PKH-26 fluorescence (red). Mice receiving miR-361-5p-repressed EPCs had more CD31+/PKH-26+ double-positive cells (white arrowheads) in ischemic muscle than another 2 control mice groups (Scr and medium). DAPI: nuclear staining of live cells (blue). (**F**) Quantitative analysis of capillary densities and CD31+/PKH-26+ double-positive cells in ischemic muscle of mice hindlimb ischemia surgery. HPF: high power field; N.D.: not detectable; *: *p*<0.05 by one-way ANOVA test followed by Tukey's post-hoc test. (**G**) A proposed model of EPC angiogenesis activities are regulated by the miR-361-5p-VEGF pathway.

Finally, we evaluated the therapeutic potential of boosting the *in vivo* vasculogenesis/angiogenesis activity of diseased EPCs by blocking miR-361-5p. Limb ischemia was induced by surgery in nude mice 3 days before CAD-EPC injection (Fig. 5A). miR-361-5p antagomirs were transfected into diseased EPCs 24 hours before transplantation, and the repression of miR-361-5p levels in transplanted EPCs at day 0 of injection were verified by RT-qPCR (Fig. 5B). Real-time PCR following showed that transfected oligos remained in CAD-EPCs even after 14 days post-transfection (*not shown*). Local EPC injection was given at the ischemic hind limb distal to the arterial occlusion site intramuscularly 3 days after the surgery, and mice were followed for 2 weeks. As illustrated in Figure 5C, mice without EPC transplantation (the EGM2 group) showed delayed blood flow recovery after ischemia surgery compared with that in mice receiving CAD-EPC (the “Scr” group, CAD-EPCs transfected with scramble oligonucleotides), as determined by Laser Doppler imaging. Meanwhile, knockdown of miR-361-5p in diseased EPCs significantly improved blood flow recovery by 90% in the ischemic limbs in mice (Figures 5C–D; n = 6 per group).

To further evaluate the effect of miR-361-5p antagomirs on the homing and differentiation to endothelial cells of the injected EPCs, as well as neovascularization/angiogenesis in mice, immunofluorescence staining was conducted on mouse tissue sample at 7 days after CAD-EPC injection. Capillaries in the ischemic muscles were visualized using anti-CD31 immunostaining (green, Fig. 5E), while injected human EPCs were detected by PKH-26 fluorescence (red, Fig. 5E). Mice that had received antagomirs transfectants showed the presence of more CD31+/PKH-26+ double-positive cells (white arrowheads) in the capillaries of the ischemic muscle compared to the medium and Scr control mice (Fig. 5E; quantitative data in Fig. 5F). The limb ischemia model showed that blocking miR-361-5p restored the defective angiogenic activities of CAD-EPCs.

## Discussion

EPCs in the systemic circulation maintain post-natal vascular homeostasis through re-endothelialization and neovascularization, and defect in EPC functions starts a cascade of events, leading to microcirculation damage, atherosclerosis and CAD [32]. The liberation of EPCs from bone marrow relies on a complex interplay between angiogenic growth factors including VEGF, chemotaxic cytokines/chemokines, potent proteases, and adhesion molecules [32], [33]. Deficiency in the autocrine VEGF expression may lead to, at least in part, the downregulation of EPC number and activity observed in a patient's peripheral blood [6], [34]. In this work, microRNAs, which may target VEGF, were predicted and among them, both microRNA-125a-5p and miR-125b-5p have been linked in inhibiting endothelin-1 expression in vascular endothelial cells [35]. MicroRNA-484 directly targets VEGFB and VEGFR2, thereby defining chemoresistance in ovarian cancer through modulation of tumor vasculature [36]. However, miR-484 did not affect VEGF levels in EPC (Fig. 3). In contrast, miR-361-5p, alone with other microRNAs known to target VEGF directly, including miR-34a, miR-503 and miR-24, were dysregulated in CAD-EPCs (Fig. 2). These miRNAs collective, and maybe synergistic, suppress the expression of endogenous VEGF, which will eventually contribute in CAD pathogenesis.

It is believed that the majority of EPCs, which may derived from the CD133+ hemangioblast stem cell population, reside in the bone marrow in a quiescent state and are mobilized into the circulation by specific stimuli. In addition to VEGF, chemotaxis factors such as granulocyte-macrophage colony-stimulating factor (GM-CSF) [37], certain drugs such as statin, ischemia, and exercise training can all liberate EPCs from the bone marrow, thereby increasing the number of circulating EPCs [38]. On top of the aforementioned autocrine stimulation scenario, serum paracrine VEGF, which is released by hypoxic/damaged tissues or cancer cells, also plays a critical role in EPC mobilization and activation. Mechanistically, circulating VEGF protein mobilizes EPCs by activating matrix metalloproteinase-9 (MMP-9), which cleaves membrane bound cKit ligand to release soluble cKit ligand (also known as stem cell factor) [39]. This then stimulates cKit+ stem cells, including EPCs, to migrate from a quiescent bone marrow niche to the vascular zone, thereby translocating the cells into a proliferative state [40]. Thus, anti-VEGF microRNAs identified in this study may contribute only in part of the reduced EPC number and activity in CAD patients. It will help to further elucidate CAD pathogenesis by identifying deregulated miRNAs suppressing cKit/CD117 and VEGF receptors (including VEGFR1/FLT1 and VEGFR2/KDR) in CAD-EPCs. For example, miR-221 and miR-222, whose expression levels are up-regulated in CAD-EPCs [41], [42], also modulate angiogenesis by targeting cKit [43].

Circulating miRNAs have been considered as biomarkers for CVDs, diabetes mellitus and cancers, and the distinct modifications in the profile of miRNAs in the blood may sometimes be detectable several years before the disease manifests [16], [29]. Recently, microRNAs are known to actively or passively released in the circulation, and the transfer of RNA *via* CD63+/CD81+ exosomes functions as a novel mode of intercellular communication [16], [44], [45]. Most of circulating microRNAs are present in human plasma and serum cofractionate with the Argonaute2 (Ago2) protein [46], [47]. However, circulating microRNAs have also been found in membrane-bound vesicles such as exosomes [45], [48]. Recent evidences point out that exosomal RNAs (exoRNAs) can be used to evaluate health status and disease progression. Moreover, circulating levels of certain miRNAs seem to be predictive of long-term complications. For example, circulating CD34(+) and CD14(+) PBMCs express and secret the antiomiR-126, and an alteration of angiomiR-126 expression in CD34(+) PBMCs in diabetes provides a novel pathway causing impaired proangiogenic effects [49]. Tumor cells also communicate with endothelial cell *via* exosomal miRNAs [50]. Here we show that miR-361-5p and miR-484 secreted by CAD-EPCs are more abundant in patient plasma (Fig. 2). Accordingly, it is possible to design new biomarker panels consisting of circulating miR-361-5p/-484 and miR-221/-222 for monitoring EPC activities *in vitro* or *in vivo* during the therapeutic procedure. Such biomarker panels will also be useful for monitoring early CVD cases among high-risk population (such as the ones with metabolic syndrome).

Although the 8 miRNAs we performed RT-qPCR validation were all up-regulated in disease EPCs, surprisingly only miR-361-5p and miR-484 were consistently more abundant in the plasma of CAD patients. Levels of five other microRNAs, especially those of miR-125b-5p and miR-34a-5p, were significantly low in patient circulation (Fig. 2). Such discrepancy may partly due to the fact that circulating miRNAs derived not only from EPCs but also other cell types such as peripheral blood mononuclear cells or smooth muscle cells. On the other hand, aberrant activity of the export machinery of EPCs under pathological conditions may also result in the abnormal expulsion of a number of microRNAs and eventually their misexpression in circulation. Exploration of the selective miRNA secretion or exosomal miRNA package in diseased EPC will help to further understanding the pathogenesis of CAD and the basic science of exosomes.

New vessel formation is controlled by a complex network of genes. Our research disclosed miR-361-5p suppresses EPC activities in CAD patients *via* targeting VEGF. With these findings, our research will lead to the development of new diagnosis and/or prognosis approaches for cardiovascular disorders as well as other EPC-related diseases such as diabetes and stroke. Novel anti-miR361-based therapeutic modalities may also be developed accordingly for targeting ischemia-related diseases. Furthermore, our findings may also be applied in treating tumor angiogenesis since the overexpression of miR-361-5p suppressed cellular migration and vasculogenesis to levels similar to those achieved by EPCs treated with the Avastin anti-VEGF mAb, a clinically used target therapy drug (Figs. 1 & 3). Because Avastin treatment is an approved therapy for metastatic colorectal cancer, metastatic kidney cancer, nonsquamous non–small cell lung cancer and glioblastoma, nucleic acid drugs such as synthetic miR-361-5p agomirs may become another choice for anti-VEGF therapy, in which its price should be cheaper than protein drugs.

## Supporting Information

Figure S1 **miR-361-5p, not miR-484, regulates EPC angiogenic activities** ***via*** **down-regulating VEGFA level.** (**A**) Overexpression of miR-361-5p in PB-EPCs enhanced angiogenesis-related activities. Upper panels, MatriGel *in vitro* tube formation assays; lower panels, Transwell cell migration assays. Quantitative results of these images are in Figure 3C. (**B**) Knock down of miR-484 by antagomir oligonucleotides in CAD-EPCs neither affected VEGF levels (upper right panel) nor increased cellular motility (lower panels).
(TIFF)

Figure S2 **VEGFA is a major downstream of miR-361-5p for regulaitng EPC acitivities.** (**A-B**) Neutralization of VEGF protein activity by the Avastin anti-VEGF mAb in CAD-EPCs transfected with miR-361-5p antagomirs repressed cellular activities. Transwell migration (**A**) and tube formation (**B**) assays were conducted and representative pictures are shown. Quantitative results of these images are in Figures 4C–D. (**C**) Addition of recombinant VEGF proteins in PB-EPC culture medium did not increase miR-361-5p expression.
(TIF)

Table S1 **Quantitative PCR primer sequences.** All primers used for determining the microRNAs or genes expression were listed in the table.
(XLSX)

Table S2 **Baseline characteristics of 55 studied subjects in healthy and CAD patients.** The criteria and clinical diagnosis for recruting subjects from either healthy or CAD patients were listed in the table. The value indicated the mean with standard deviation or number with percentage.
(XLSX)
