# Supplementary figures and images for: Dysregulated miR-361-5p/VEGF Axis in the Plasma and Endothelial Progenitor Cells of Patients with Coronary Artery Disease
Source: PLoS One. 2014 May 27;9(5):e98070. doi: 10.1371/journal.pone.0098070 (PMC4035317; doi:10.1371/journal.pone.0098070)

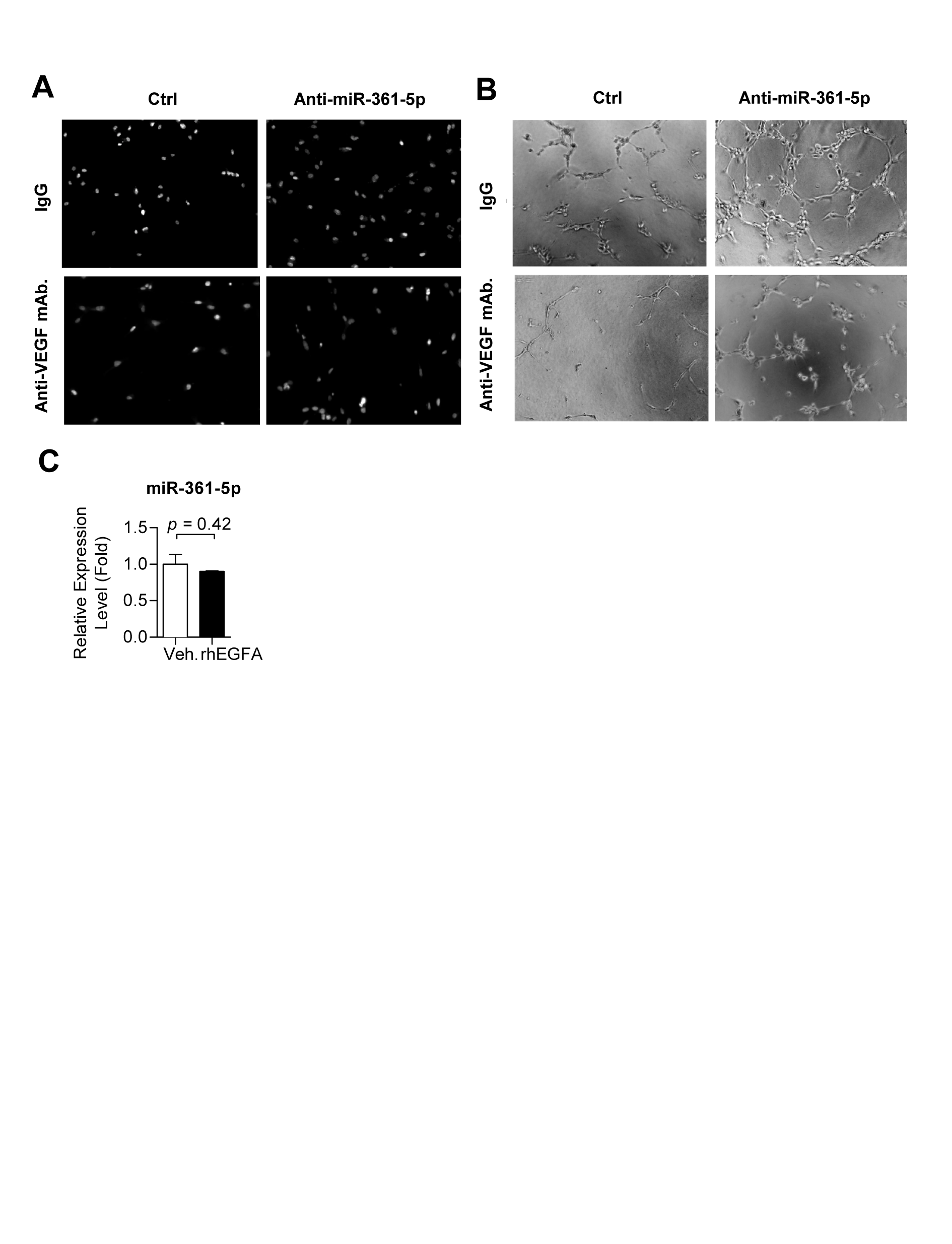

Supplement: Figure S2 — VEGFA is a major downstream of miR-361-5p for regulaitng EPC acitivities. (A-B) Neutralization of VEGF protein activity by the Avastin anti-VEGF mAb in CAD-EPCs transfected with miR-361-5p antagomirs repressed cellular activities. Transwell migration (A) and tube formation (B) assays were conducted and representative pictures are shown. Quantitative results of these images are in Figures 4C–D. (C) Addition of recombinant VEGF proteins in PB-EPC culture medium did not increase miR-361-5p expression. (TIF) [file pone.0098070.s002.tif]
